# Systemic lupus erythematosus as the paradigm for understanding the complex immune relationships and therapeutic opportunities for targeting complement in autoimmune diseases^☆^

**DOI:** 10.1016/j.imbio.2025.152915

**Published:** 2025-05-20

**Authors:** V. Michael Holers

**Affiliations:** Division of Rheumatology, University of Colorado School of Medicine, Aurora, CO, USA

**Keywords:** Therapeutics, Autoimmune Disease, Biomarkers, Animal Models, Clinical Trials

## Abstract

Complement therapeutics have been increasingly tested and approved for human diseases, often in orphan diseases with strong and apparently causal genetic linkage or mutation-associated features. However, the complement system has been demonstrated to be activated in essentially all human inflammatory, ischemic and autoimmune diseases, suggesting the possibility of even wider therapeutic applications. The goal of this manuscript is to review some of the evidence supporting a wide role for complement in the specific treatment of autoimmune diseases, especially as recent approvals in autoantibody-driven diseases are opening the door to others of these indications. However, in part because of a dearth of complement biomarker data obtained during clinical trials, it is not known what findings would help to predict therapeutic success in other autoimmune diseases. To frame the discussion, it is relevant to point out that the disease systemic lupus erythematosus (SLE) has been among the most extensively studied autoimmune disease with regards to the varied roles of the complement system, and there are available both human phenotypic studies and murine model data. Because of that history, SLE will be focused upon herein, the many roles of complement in SLE will be reviewed, and informative comparisons to other autoimmune diseases will be made. In aggregate, experimental and phenotypic data suggest that each human autoimmune disease deserves careful attention to the possibility that a specific complement inhibitor targeting the most relevant complement convertase or component will be of benefit, and thus therapeutic approaches should be tested using informative biomarker-driven clinical trial strategies.

## 1. Introduction

In science, a paradigm can constitute a distinct set of concepts that centrally contribute to the understanding of a field. The word is Greek in origin and is often used to provide a framework in which broad activities are presented and discussed. This review aims to explore the many activities of the complement system and how they contribute to the development and pathogenesis of human autoimmune diseases. Within that broad context, the disease systemic lupus erythematosus (SLE) has long been extensively studied with regards to the many varied roles of complement in disease risk, immune dysregulation and organ damage (Manderson et al., 2004). Because of that history, it is a fitting disease upon which to focus. Thus, the primary effort herein will be to review how the specific activities of complement that have been studied in SLE and its animal models serve as paradigms around which to understand the broader roles of this intriguing pathway in the immunopathogenesis of other autoimmune diseases and its therapeutic potential (Ricklin et al., 2018). As a direct extension of these studies, working to modulate deleterious activities in SLE and other autoimmune diseases through the incorporation into human studies of informative biomarkers (Nilsson and Ekdahl, 2012) as well as testing the increasing number of approved complement therapeutics (West et al., 2024) in patients in a mechanism-driven manner remain promising areas for further exploration.

### 1.1. Knowledge gained from SLE studies can be used to predict roles of complement in other autoimmune diseases

SLE is a complex female predominant disorder with an incidence of 5–12/100,000 person-years and extensive interactions with components of the complement system (Fanouriakis et al., 2021; Li et al., 2021; Siegel and Sammaritano, 2024). The disease process is characterized by immunologic features, including dysregulation of innate and adaptive immunity, the former characterized in part by continuous neutrophil activation and the latter by the presence of a wide variety of autoantibodies. SLE is generally considered to be a systemic disorder, but with immune-mediated damage that can affect almost every organ system, most often renal (designated lupus nephritis), mucocutaneous, musculoskeletal, serosal, hematologic and neuropsychiatric (Siegel and Sammaritano, 2024). Current therapeutic approaches in SLE include glucocorticoids, hydroxychloroquine, immunosuppressives (cyclophosphamide, azathioprine, mycophenolate mofetil, voclosporin), and targeted biologics (belilumab, anifrolumab, rituximab), as well as additional off label medications when specific clinical situations arise (Siegel and Sammaritano, 2024).

**Fig. 1.**
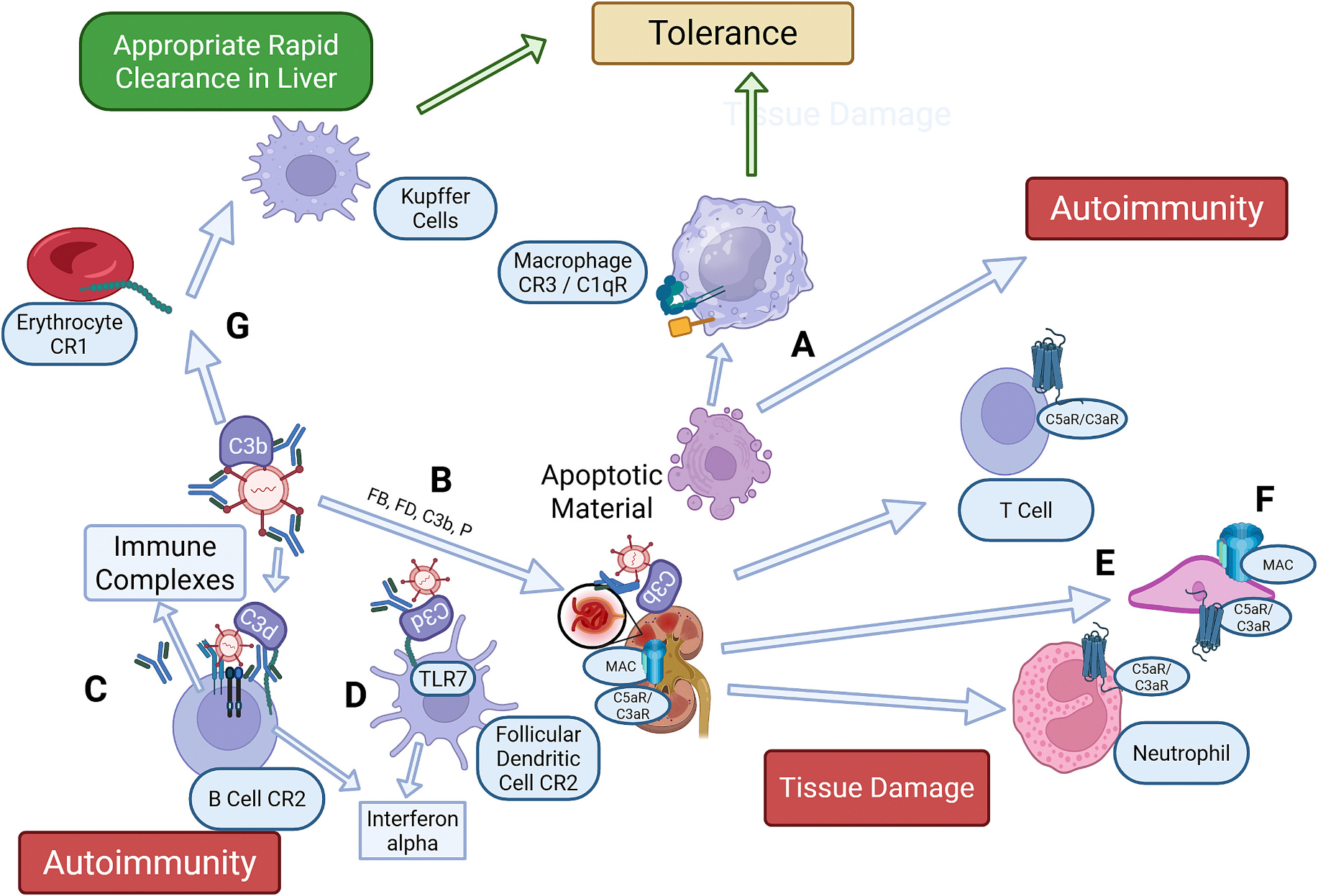
Illustration of the many roles of the complement system in the development of SLE, and by inference other autoimmune diseases. (A) Apoptotic material if appropriately cleared through interactions with C1q and its receptor will lead to tolerance, and if not will lead to autoimmunity; (B) In complement activation, the alternative pathway is necessary in SLE and some settings to lead to tissue damage, for instance in the kidney where immune complexes also deposit; (C) CR2 can amplify B cell responses to both foreign and apparently self antigens; (D) CR2 on B cells and follicular dendritic cells amplifies interferon-alpha production; Tissue injury and modulation thereof occurs through complement system C5aR1 and C3aR (E), as well the MAC (F); (G) Appropriate clearance of circulating immune complexes in SLE requires C3b-coated immune complexes to bind to CR1 on erythrocytes and be transported for clearance in the liver. Created in BioRender. Holers, V. (2025) https://BioRender.com/ mm8szox.

The complement system is understood to play several integral roles in the pathogenesis of SLE, both with regards to risk of development of the disease (Botto et al., 2009) as well as through the engagement of potent effector mechanisms once clinical disease is established (Fig. 1) (Li et al., 2021). These roles have been primarily explored through genetic, biomarker, animal model, and *ex vivo* studies, as well as in some emerging clinical trials.

### 1.2. Historic and next generation complement genetic studies have contributed to understanding of the risk of SLE and other autoimmune diseases

Although complement effector functions are often considered to be most relevant to lupus clinical manifestations, deficiencies of classical pathway (CP) proteins remain among the most impactful genetic associations with SLE (Macedo and Isaac, 2016). Specifically, although individual studies present varied levels of association, development of SLE is highly associated with complete deficiencies of C1q (90–95 %) and C4 (75 %). Additional associations with SLE are found with complete deficiencies of C1r/C1s (60–66 %) and C2 (~10 %). Perhaps the most fascinating association with SLE occurs with the C4 genes *C4A* and *C4B*, which exhibit substantial variation in risk for SLE, with *C4A* protecting more strongly than *C4B*, but also very impressive sex-biased risk in their effects (Kamitaki et al., 2020). In that situation, C4 alleles appear act more strongly in men than in women when assessing risk for SLE as well as the disease schizophrenia. With regards to complement regulatory proteins, the genetics of the factor H (FH) and FH-related (FHR) family have been studied in patients with SLE, with the finding that the *FH* locus is linked to SLE risk (Zhao et al., 2011) and also that the relatively common linked deletion of *FHR3* and *FHR1* genes contributes an elevated risk to the development of SLE across multiple ethnic populations (Zhao et al., 2011). Although the mechanisms of effects of complement gene variants in patients are unknown, they are often considered to reflect defects in clearance of immune complexes and apoptotic bodies (Botto and Walport, 2002), promoting loss of self-tolerance and inappropriate tissue deposition (Fig. 1A). However, more recent experimental models have suggested that the complete C1q and the *C4A*/*C4B* polymorphic variant effects regulate the development of loss of self-tolerance through effects on CD8 T cell metabolism (Ling et al., 2018) and autoantibody producing B cell development (Simoni et al., 2020), respectively. The mechanism of effects of the *FHR3*/*FHR1* deletion are unknown, but are likely due to as yet uncharacterized immunomodulatory effects.

Acquired alterations in C1q functions are also found in patients, typically associated with anti-C1q autoantibodies that are present in an especially high proportion of patients with lupus nephritis (Beurskensa and van Schaarenburgb, 2015). Other acquired associations include the loss of complement receptor 1 (CR1/CD35) from the surface of erythrocytes that is apparently secondary to excessive levels of C3 fragment-coated immune complex clearance and cleavage of CR1 in the liver (Schifferli et al., 1989). Although the associations of complement deficiencies with SLE are most often commented upon in publications, it is relevant to emphasize that patients with CP deficiencies are also at risk for severe infections that can be fatal (Macedo and Isaac, 2016).

With this extensive background in SLE, other autoimmune diseases have also demonstrated substantial genetic associations with the complement pathway (Coss et al., 2024; Jia et al., 2022). The most impactful associations are gene mutations, often found in the renal diseases atypical hemolytic uremic syndrome (aHUS) and C3 glomerulopathy (C3G), present in the alternative pathway (AP) activation and regulatory proteins, as well as inhibitory autoantibody generation (Noris et al., 2010). Other intriguing associations are found with the FHR family (Poppelaars et al., 2021a), where as opposed to the deletion of *FHR3*/*FHR1* being associated with a higher risk of SLE, there is a lower risk of developing IgA nephropathy (IgAN) (Gharavi et al., 2011). Clinically useful next generation sequencing approaches are now available to evaluate patients with these thrombotic microangiopathies (TMA) and other potential complement-related renal diseases (Java and Kim, 2023). Importantly, the use of these approaches more broadly in autoimmune diseases should increase our understanding of disease pathogenesis and the potential for complement pathway therapeutic intervention.

### 1.3. Biomarkers of complement activation are readily detected in patients with SLE and other autoimmune diseases

Complement biomarkers can be used to discern the functional and activation status of specific activation pathways as well as common effector mechanisms (Nilsson and Ekdahl, 2012). Examples include C1, C4 and C2 for the CP, factor B (FB) and factor D (FD) for the AP, and ficolins, mannose-binding lectin (MBL) and MBL-associated serine proteases (MASPs) for the lectin pathway (LP). Activation products include C4d (CP and LP), and Bb and Ba (AP), while other effector mechanism components [C3a, C5a and C5b-9/membrane attack complex (MAC)] are products of the common pathway that follows the initiation mechanisms. Additional biomarkers consisting of measures of the intact multi-protein convertases are also increasingly available.

Many informative biomarkers have been studied in patients with SLE. For instance, while low C3, C4 and total hemolytic complement (THC) are most often associated with active disease, the plasma levels of FH are inversely associated with clinical disease activity scores and positively associated with serum C3 levels, reflecting likely decreases in complement activation (Wang et al., 2012). Whether this kind of FH/C3 level relationship exists for diseases without systemic activation is not clear. Additionally, there is an inverse relationship between the injury scores in lupus renal biopsies, including those patients with TMA, and serum FH levels. When comparing the levels of complement activation factors in SLE patients with glomerulonephritis as compared to those without this target organ involvement, plasma levels of Bb, C3a, C5a and sC5b-9 were all significantly increased, and Bb levels were the most highly associated with worse outcomes and renal pathology scores. Beyond this, the ratio of iC3b to C3 was found to be increased as a biomarker of elevated disease activity (Kim et al., 2019), and evidence of AP activation acted as an antecedent predictor of future flares (Buyon et al., 1992). In addition to blood levels of biomarkers, renal mRNA expression has been studied in the kidneys of patients with proliferative lupus nephritis, pre- and post-treatment for a renal flare (Parikh et al., 2017). Notably, in therapeutic non-responders complement *C3* and *FD* mRNA levels were substantially elevated, while mRNA from the *FI* gene was decreased. In addition to mRNA levels, the presence of both FH and FB proteins in the glomerulus were associated with interstitial fibrosis, and those biopsies with localized properdin (P) exhibited higher proteinuria (Sato et al., 2011). Not surprisingly, the presence of C3 fragments in the first renal biopsy is associated with the doubling of serum creatinine in the future (Hill et al., 2001). Beyond the kidney and blood findings, even in early classic studies complement C3 fragments were found to be associated with local IgG deposition in patients with SLE in the liver, spleen, heart and other organs (Walport, 2002; Paronetto and Koffler, 1965). More recently, covalently cell-bound C3d and C4d have been identified in patients with SLE on erythrocytes and other circulating cell types, and the relative levels exhibit the potential to portend disease flares (Ramsey-Goldman et al., 2017). In sum, the systemic and tissue-specific autoimmunity in SLE is associated with widespread evidence of complement dysregulation and deposition.

With regards to complement elevations in other autoimmune diseases, many diseases are characterized by the presence of activation in the specific target organs (Coss et al., 2024; Jia et al., 2022). Perhaps the most relevant to review are the autoimmune diseases in which complement therapeutics have been approved. These include cold agglutinin disease (CAD), generalized myasthenia gravis (gMG), neuromyelitis optica (NMO), and anti-neutrophil cytoplasmic antibody (ANCA)-associated vasculitis (AAV).

With regards to CAD, the disease is typically characterized by cold-reactive IgM autoantibodies of monoclonal origin that function as hexamers, and an associated anemia that has been considered for decades to be due to the clearance of IgM and CP derived C3 fragment-bound cells by complement receptors in the reticuloendothelial system in a process designated extravascular hemolysis (Shi et al., 2014). Recent clinical trials using the C1s monoclonal antibody (mAb) inhibitor sutimlimab were successful in reducing hemolysis and improving fatigue, and led to the approval of this drug in patients with CAD (Röth et al., 2021). With regards to complement biomarkers, C4 levels are often decreased due to the presumed CP consumption as it activates C3 as the C3b fragment on the erythrocyte surface, and the “surviving” erythrocytes are found to be coated with the terminal C3d cleavage fragment after the surface C3b is proteolytically processed to this fragment (Berentsen et al., 2022).

With regards to gMG, the pathogenesis of experimental MG has been studied for decades and shown to involve anti-acetylcholine receptor antibodies that are present in the neuromuscular junction (Nakano and Engel, 1993). These autoantibodies fix complement through the CP and cause neuromuscular dysfunction in experimental models, primarily through MAC formation (Morgan et al., 2006). More recently, complement biomarkers have been measured in patients with gMG, demonstrating that C2 and C5 levels are significantly reduced, and C3, C3b and C5a increased (Iacomino et al., 2022). Beyond these findings, gMG patients have demonstrated higher plasma C3a and soluble C5b-9, as well as correlations of a composite disease severity score with levels of plasma FB, FI, and FH Huang et al., 2024. With this extensive experimental background, the finding that ravulizumab demonstrated clinical benefit sufficient for approval was not unexpected (Vu et al., 2023).

Another condition with extensive connections to complement is NMO, a disease associated with IgG autoantibodies directed to aquaporin 4 that typically presents with transverse myelitis and optic nerve dysfunction (Wingerchuk and Lucchinetti, 2022). In a clinical trial, eculizumab demonstrated a significant decrease in relapse rates (Pittock et al., 2013), which led to regulatory approval. Local evidence of complement activation in the central nervous system is provided by findings of elevations of C3a and C5a in the cerebrospinal spinal fluid as well as MAC deposition within the NMO lesions (Bennett and Owens, 2017). Circulating activation products from the classical (C4d, iC3b), alternative (Bb, iC3b) and terminal pathways (C5a, sC5b-9) are elevated in patients (Hakobyan et al., 2017).

The fourth autoimmune disease example is provided by AAV, a potentially life threatening disorder characterized by small vessel inflammation, endothelial dysfunction, and related organ complications (Kitching et al., 2020). Patients with subtypes of AAV exhibit variable rates of autoantibodies to proteinase 3 (PR3) and/or myeloperoxidase (MPO), which are neutrophil cytoplasmic proteins. Significant clinical benefit has been shown to occur in clinical trials using the C5aR1 inhibitor avacopan in AAV, where it was paired with rapid glucocorticoid decreases (Jayne DRW, Merkel PS, Schall TJ, Bekker P, Group AS, 2021), resulting in approval in this patient population.

With regards to complement biomarkers in AAV, deposition of C3 fragments and MAC has been found in the kidney, as well as significant elevations of circulating C3a, C5a and sC5b-9 whose levels correlate with disease activity (Wu et al., 2019; Kallenberg and Heeringa, 2012). In addition, an increase in circulating C5a has been detected prior to the onset of a clinical flare (Johansson et al., 2022). In histologic studies, the presence of renal Bb correlates with crescents, interstitial infiltrates and fibrosis, as well as tubular atrophy (Gou et al., 2013). Beyond these sites, urinary levels of Bb correlate with the serum creatinine, and the presence of renal C3 fragments is associated with worse disease outcomes (Oba et al., 2021).

### 1.4. Functions of the complement system likely involved in the pathogenesis of SLE and other autoimmune diseases

From experimental models and clinical studies, the complement system is known to affect many aspects of the innate and adaptive immune systems. Here these various activities are described, initially as they have been studied in SLE but also providing examples of similar roles in other autoimmune diseases.

#### Complement Activation Pathways and the Key Role of the Alternative Pathway and Amplification Loop.

The clinical success of therapeutics targeting the classical pathway, as well as the C3/C5 convertases, the AP and C5aR1, has provided substantial impetus to understanding the mechanisms by which complement activation is initiated and regulated in human disease states. Certainly the success of C1s-targeted CP inhibition in CAD, and nascent studies of C2 inhibition in experimental models and patients with neurologic diseases such as multifocal motor neuropathy (Budding et al., 2022) indicates that IgM and complement fixing isotypes of IgG can play important roles in human autoimmune diseases (Gewurz et al., 1995). In contrast, LP initiation through the binding to ligands by mannose-binding lectin (MBL) and activation of the MASPs plays a less certain role (Reid and Turner, 1994; Matsushita et al., 2000).

In contrast, AP auto-activation through “tickover” has the capacity to be engaged in many settings (Muller-Eberhard, 1988; Elvington et al., 2019). Regardless of the nature of the initiators, all three activation pathways will generate C3b molecules that will engage the amplification loop. Although the absence of published trials in patients with SLE limits understanding of the role of the AP in this disease, murine models have been extensively studied to evaluate mechanisms potentially involved in the development of the human disease, including lupus nephritis (McGaha and Madaio, 2014). In this setting, although SLE is considered to be an autoantibody driven disease, support for the essential role of the AP and amplification loop was provided by the finding that *FB*−/− MRL/*lpr* mice demonstrated substantial protection from development of nephritis (Fig. 1B) (Watanabe et al., 2000). Similarly, *FD*−/− MRL/*lpr* mice lacking the key AP protein FD were protected (Elliott et al., 2004). Subsequently, another AP inhibitor, CRIg-Fc, was also found to ameliorate development of glomerulonephritis in MRL/*lpr* mice (Lieberman et al., 2015). These findings are in contrast to findings that *C3*−/− mice were not protected in this model (Sekine et al., 2001), and that *C4*−/− mice in a separate model of SLE demonstrated enhanced autoimmunity (Einav et al., 2002).

Following upon the results in murine models of SLE, the role of the AP has been explored in a number of other autoimmune diseases. Perhaps most relevant is IgA nephropathy (IGAN), the most common form of primary glomerulonephritis in the world (Penfold et al., 2018). Therein, recent clinical trials and approvals have provided great insights into the roles of complement, in particular the C3/C5 convertase (Dixon et al., 2023) and AP (Perkovic et al., 2025), the latter leading to approval of the small molecule FB inhibitor iptacopan. The pathogenesis of IgAN appears to be a systemic disorder that targets the kidneys (Suzuki et al., 2011). Renal histologic features include immune complexes containing galactose-deficient IgA1 (Gd-IgA1), IgG and C3 activation fragments. In patients, elevated circulating C3b, iC3b and C3dg are found in ~30 % of patients, and renal biopsies typically contain FH, FB and P (Le Stang et al., 2021; Poppelaars et al., 2021b). The role of the AP remains to be explored in other autoimmune disorders associated with and/or driven by autoantibodies. However, hope for beneficial effects comes from various animal models, for instance AAV where *FB*−/− mice are protected in the highly informative murine model of this disease (Xiao et al., 2007), and the CAIA model of RA, where similarly *FB*−/− but not *C4*−/− mice are protected from the development of arthritis (Banda et al., 2006).

#### Regulation of B and T Cell Autoimmune Responses, as Well as Effector Functions, by Complement Activation Fragments and Their Receptors.

In addition to the effects of C1q discussed above, there are other well-defined intersections between complement activation fragments and the adaptive immune system. One consists of the complement receptor type 2 (CR2/CD21), which is most highly expressed on B cells and follicular dendritic cells. CR2 plays a key role in the development of high affinity antibodies and long-lasting memory to foreign antigens (Carroll and Isenman, 2012). When CR2 is bound by its primary C3 activation fragment-derived ligand, designated C3d, it co-associates with CD19 on B cells to amplify B cell receptor (BCR) signaling. C3d and CR2 also mediate immune complex binding to follicular dendritic cells.

As the development of SLE involves subversion of normal B cell tolerance checkpoints, it was expected that CR2 ligation by C3d-bound immune complexes would promote the development of autoantibodies and a worsened outcome in models of SLE. However, prior studies in murine models of SLE using gene-targeted *Cr2*−/− mice, which lack both CR2 and complement receptor 1 (CR1/CD35), demonstrated variable results, possibly due to the associated lack of interactions by C4b itself with CR1 being a dominant effect (Einav et al., 2002). As an alternate approach to address this question, a highly specific mouse anti-mouse C3d monoclonal antibody was created that blocks interaction with CR2 (Thurman et al., 2013). With this tool, disruption of the critical C3d-CR2 ligand-receptor binding step alone substantially delayed autoimmunity and renal disease progression in the MRL/*lpr* model of SLE (Fig. 1C) (Kulik et al., 2019).

Recent data have also highlighted another potential role for CR2 in its interactions with targets. One study suggested that this receptor could also interact with DNA and result in interferon alpha, a major cytokine driver of SLE pathogenesis, release (Fig. 1D) (R. A, Banda N, Szakonyi G, Chen XS, Holers VM., 2013). A second suggested that engagement of CR2 on follicular dendritic cells would allow a TLR7-dependent mechanism to be triggered, resulting in production by these cells of interferon alpha (Fig. 1D) (Das et al., 2017).

With regards to other models, CR2 engagement has been found to be essential for the development of the human rheumatoid arthritis (RA) model collagen-induced arthritis (CIA) (Kuhn et al., 2008) and experimental autoimmune myocarditis (Kaya et al., 2001). Unfortunately, this target has not been explored yet in patient studies.

Beyond CR2, C5a and C3a have been most often considered as a targets due to their effects on promoting innate immune-generated inflammation (Wetsel, 1995; Pandey et al., 2020). C5a exhibits its immunoregulatory properties through its primary receptor designated C5aR1/CD88, through which it will promote chemotaxis, neutrophil activation and platelet degranulation, among other largely pro-inflammatory effects (Fig. 1E). Prior studies have demonstrated, however, that C5aR1 and C5aR2 can also affects adaptive immune responses (Kohl, 2006). C3a manifests its effects through its C3aR and can typically play in experimental models either pro-inflammatory or- immunomodulatory roles (Humbles et al., 2000; Gao et al., 2020). Studies in the MRL/*lpr* model with C5aR1 deficient animals led to prolonged survival, less renal disease and decreased autoantibodies (Wenderfer et al., 2005). Conversely, MRL/*lpr* mice deficient in C3aR demonstrated accelerated renal disease and increased autoantibody generation, but no effects on survival (Wenderfer et al., 2009).

With regards to other autoimmune disease models, C5aR1 has been successfully targeted in patients with AAV, and it has also been studied in many other autoimmune disease models. These include collagen antibody-induced arthritis (Banda et al., 2012), anti-phospholipid antibody-induced fetal loss (Girardi et al., 2003) and epidermolysis bullosa acquisita, although in this syndrome both C5aR1 and C5aR2 appear to be involved in promoting pathogenesis (Seiler et al., 2022). Thus, this ligand and receptor could well be effective in diseases in which adaptive immunity and complement effector functions are co-drivers of organ damage.

#### Engagement of Pro-Inflammatory Pathways Through the Membrane Attack Complex.

Although the MAC is most often considered in the context of cell lysis, it has been known for decades to deposit on nucleated cells and initiate a large number of activation events that are typically pro-inflammatory and can disrupt tissue homeostasis (Morgan et al., 2017). With regards to SLE, although experimental C5 inhibition has long been known to ameliorate the (NZBxNZW)F_1_ experimental model (Wang et al., 1996), and as shown above the C5a product of C5 activation promotes renal injury (Wenderfer et al., 2005), the role of the MAC itself beyond C5a does not appear to have been studied in either patients or published models through inhibitory approaches (Fig. 1F). However, another way to understand the role of the MAC is through gene targeting of the MAC inhibitor CD59, where for example the lack of expression accelerated autoimmunity and target organ damage in the MRL/*lpr* model (Miwa et al., 2012).

Perhaps the best studied example demonstrating a role for the MAC in an autoimmune disease comes from studies of experimental multiple sclerosis and patients with this disease (Morgan et al., 2021; Morgan and Harris, 2015). Specifically, the brains of patients demonstrate extensive immune infiltration, local production of complement factors, and complement activation. In experimental models, inhibition of proximal activation steps can ameliorate models; however, these are relatively unique in demonstrating through specific inhibition approaches a role for the MAC in disease pathogenesis. In addition, when using the CD59 knockout approach, a rat passive transfer model of NMO was greatly accentuated in deficient mice (Yao and Verkman, 2017).

#### Clearance of Circulating Immune Complexes.

In addition to the effects of complement on innate and adaptive immunity, a number of important inter-linked “housekeeping” functions are undertaken by this system. One important function is the clearance from the circulation of circulating immune complexes, most often containing IgG antibodies and infectious organisms or their antigens through binding to erythrocyte CR1, in this activity known as the immune adherence receptor (Fig. 1G) (Miyakawa et al., 1981; Nelson, 1953). This function is very much diminished in patients with SLE, as noted above, and also dysfunctional in other systemic diseases such as large vessel vasculitis associated with mixed essential cryoglobulinemia, which is characterized by excessive immune complex formation and their presence in the circulation and abnormal tissue deposition (Schifferli and Taylor, 1989).

#### Extrinsic Complement Activation Mechanisms.

Although not extensively studied in SLE, mention of other mechanisms of complement activation is relevant. One of the most important is the extrinsic pathway that is primarily initiated by a number of proteases that are generated in disease settings (Mastellos and Lambris, 2025). A recent example is the observation that granzyme K can cleave C4 and C2 to create the CP/LP C3 convertase C4b/2a Donado et al., 2025.

### 1.5. Thoughts on creating more informed therapeutic strategies to facilitate broadening the use of complement targeted drugs in autoimmune diseases

Despite increasing therapeutic benefits being demonstrated in autoimmune diseases, what is lacking in the field is a comprehensive approach to collecting and publishing results of complement-related biomarkers (Mastellos et al., 2019). That is especially relevant when there are pro-inflammatory mediators from this pathway whose levels are intended to be modulated through therapeutic intervention, and when there is a need to understand the specific relationships of such biomarker changes to the relative improvement in a patient clinical status. Such studies would also provide important confirmation for the field that the intended effects are found when undertaking a targeted therapy, for instance demonstrating in a disease setting that FB cleavage products Ba and/or Bb are altered to the expected level by inhibitors of FB or FD. One such informative approach was recently illustrated by the finding that alternative pathway inhibition of FB by the small molecule iptacopan resulted in the expected decrease in C3 fragment binding to red blood cells (Peffault de Latour et al., 2024) in patients with paroxysmal nocturnal hemoglobinuria (PNH), which was an effect found when patients are treated only with C5 inhibition and C3 activation continues unabated (Risitano, 2012). Without this knowledge in general, there remain many questions such as whether it requires essentially 100 % inhibition to see maximal clinical benefit, which is apparently the requirement for targeting C5 in PNH Gaya et al., 2023. In addition, as many diseases are focused in tissues and organs, and not peripheral blood where samples are relatively accessible, it is unknown as to whether control of complement activation in tissue samples reflects the changes seen in peripheral blood, or other fluids such as urine. Thus, linking immunohistochemistry of organ biopsies to assess changes in local complement activation to tissue damage to peripheral blood biomarkers is much needed in the field. *“*Beyond these questions, other considerations arising from such studies would potentially support the use of combination therapy, for instance to block AP activation in addition to C5aR1 engagement, with the goal to block the deleterious effects of C3 activation in addition to potential C5a effects through extrinsic activation or specific effects on neutrophils as seen in AAV (Jayne DRW, Merkel PS, Schall TJ, Bekker P, Group AS, 2021).

Additional approaches to understanding the level of local control of complement activation in target organs are emerging. One organ that is of particular interest is the kidney, as one can biopsy this organ with reasonable safety and thus directly compare results from tissue samples to those derived from emerging minimally invasive imaging approaches. The ability to specifically detect and quantitate complement *in situ* using an imaging approach was recently shown by the ability of a mAb specific for C3d/iC3b to detect renal deposition of the specific target (Renner et al., 2023). As the field expands to other organs and other complement pathway targets, it is anticipated that the ability to image key activation fragments such as C3d, C4d and the MAC, as well as receptors such as C5aR1 will also have to expand in order to provide additional information about the extent and location(s) of activation, and assure target coverage at the site(s) of interest.

## 2. Conclusions

The complement pathway is activated in many if not all autoimmune diseases. As several autoantibody-driven autoimmune diseases have been shown to benefit from C5 or FB inhibition, it is likely that many others will exhibit similar amelioration following treatment with inhibitors that target specific proteins or convertases. Although patients with SLE, and especially lupus nephritis, have not yet been shown to benefit from a complement inhibitor, some trials are underway. Regardless, this disease has been extensively studied to understand the roles of complement in disease risk, adaptive autoimmunity, evolution and organ damage. Thus, like other diseases, it is hoped that patients with at least some form of SLE will similarly benefit. There are additional studies that would improve the likelihood of successful outcomes, mostly focused on improving the use and utility of complement biomarkers, including molecular imaging approaches.

## Data Availability

No data was used for the research described in the article.

## Glossary

SLE: systemic lupus erythematosus
FH: factor H
FHR: factor H-related
C3G: C3 glomerulopathy
IGAN: IgA nephropathy
TMA: thrombotic microangiopathy
THC: total hemolytic complement
CAD: cold agglutinin disease
NMO: neuropmyelitis optica
ANCA: anti-neutrophil cytoplasmic antibody
AAV: ANCA-associated vasculitis
PR3: proteinase 3
MPO: myeloperoxidase
FB: Factor B
MBL: mannose-binding lectin
MASP: (MBL-associated serine protease)
FD: factor D
P: properdin
BCR: B cell receptor
CR2: complement receptor 2
CR1: complement receptor 1
CR3: complement receptor 3
MAC: membrane attack complex
PNH: paroxysmal nocturnal hemoglobinuria

## References

[R1] Banda NK, Thurman JM, Kraus D, , 2006. Alternative complement pathway activation is essential for inflammation and joint destruction in the passive transfer model of collagen-induced arthritis. J. Immunol. 177, 1904–1912. 16849503 10.4049/jimmunol.177.3.1904

[R2] Banda NK, Hyatt S, Antonioli AH, , 2012. Role of C3a receptors, C5a receptors, and complement protein C6 deficiency in collagen antibody-induced arthritis in mice. J. Immunol. 188, 1469–1478. 22205026 10.4049/jimmunol.1102310 PMC3262949

[R3] Bennett JL, Owens GP, 2017. Neuromyelitis optica: deciphering a complex immune-mediated astrocytopathy. J. Neuroophthalmol. 37, 291–299. 28410278 10.1097/WNO.0000000000000508 PMC5557670

[R4] Berentsen S, Berentsen S, D’Sa S, Randen U, Małecka A, Vos JMI, 2022. Cold agglutinin disease: improved understanding of pathogenesis helps define targets for therapy. Hemato 3, 574–594.

[R5] Beurskensa FJ, van Schaarenburgb RA, 2015. L.A. T. C1q, antibodies and anti-C1q autoantibodies. Mol. Immunol. 68, 6–13. 26032012 10.1016/j.molimm.2015.05.010

[R6] Botto M, Walport MJ, 2002. C1q, autoimmunity and apoptosis. Immunobiology 205, 395–406. 12396002 10.1078/0171-2985-00141

[R7] Botto M, Kirschfink M, Macor P, Pickering MC, Wurzner R, Tedesco F, 2009. Complement in human diseases: Lessons from complement deficiencies. Mol. Immunol. 46, 2774–2783. 19481265 10.1016/j.molimm.2009.04.029

[R8] Budding K, Johansen LE, Van de Walle I, , 2022. Anti-C2 antibody ARGX-117 inhibits complement in a disease model for multifocal motor neuropathy. Neurol. Neuroimmunol. Neuroinflamm. 10.1212/NXI.00000000000011. PMC8587732 34759020

[R9] Buyon JP, Tamerius J, Ordorica S, Young B, Abramson SB, 1992. Activation of the alternative complement pathway accompanies disease flares in systemic lupus erythematosus during pregnancy. Arthritis Rheum. 35, 55–61. 1731815 10.1002/art.1780350109

[R10] Carroll MC, Isenman DE, 2012. Regulation of humoral immunity by complement. Immunity 37, 199–207. 22921118 10.1016/j.immuni.2012.08.002 PMC5784422

[R11] Coss SL, Zhou D, Chua GT, , 2024. The complement system and human autoimmune diseases. J. Autoimmun. 137. 10.1016/j.jaut.2022.102979 PMC10276174 36535812

[R12] Das A, Heesters BA, Bialas A, , 2017. Follicular dendritic cell activation by TLR ligands promotes autoreactive B cell responses. Immunity 46, 106–119. 28099860 10.1016/j.immuni.2016.12.014 PMC8140609

[R13] Dixon BP, Greenbaum LA, Huang L, Rajan S, Ke C, Zhang Y, 2023. Clinical safety and efficacy of pegcetacoplan in a phase 2 study of patients with C3 glomerulopathy and other complement-mediated glomerular diseases. Kid. Intl. Rep. 8, 2284–2293. 10.1016/j.ekir.2023.08.033 PMC10658235 38025230

[R14] Donado CA, Theisen E, Zhang F, , 2025. Granzyme K activates the entire complement cascade. Nat. Immunol. 641, 211–221. 10.1038/s41586-025-08713-9 PMC12180478 39914456

[R15] Einav S, Pozdnyakova OO, Ma M, Carroll MC, 2002. Complement C4 is protective for lupus disease independent of C3. J. Immunol. 168, 1036–1041. 11801636 10.4049/jimmunol.168.3.1036

[R16] Elliott MK, Jarmi T, Ruiz P, Xu Y, Holers VM, Gilkeson GS, 2004. Effects of complement factor D deficiency on the renal disease of MRL/lpr mice. Kidney Int. 65 (1), 129–138. 14675043 10.1111/j.1523-1755.2004.00371.x

[R17] Elvington M, Liszewski MK, Liszewski AR, , 2019. Development and optimization of an ELISA to quantitate C3(H2O) as a marker of human disease. Front. Immunol. 10. 10.3389/fimmu.2019.00703 PMC6458276 31019515

[R18] Fanouriakis A, Tziolos N, Bertsias G, Boumpas DT, 2021. Update οn the diagnosis and management of systemic lupus erythematosus. Ann. Rheum. Dis. 80, 14–25. 33051219 10.1136/annrheumdis-2020-218272

[R19] Gao S, Cui Z, Zhao M. h., 2020. The complement C3a and C3a receptor pathway in kidney diseases. Front. Immunol. 10.3389/fimmu.2020.01875. PMC7461857 32973774

[R20] Gaya A, Munir T, Urbano-Ispizua A, , 2023. Results of a phase 1/2 study of cemdisiran in healthy subjects and patients with paroxysmal nocturnal hemoglobinuria. EJHaem 4, 612–624. 37601837 10.1002/jha2.748 PMC10435727

[R21] Gewurz H, Zhang XH, Lint TF, 1995. Structure and function of the pentraxins. Curr. Opin. Immunol. 7 (1), 54–64. 7772283 10.1016/0952-7915(95)80029-8

[R22] Gharavi AG, Kiryluk KK, Choi M, , 2011. Genome-wide association study identifies susceptibility loci for IgA nephropathy. Nat. Genet. 13. 10.1038/ng.787 PMC3412515 21399633

[R23] Girardi G, Berman J, Redecha P, , 2003. Complement C5a receptors and neutrophils, but not activating Fcg receptors, are critical mediators of anti-phospholipid antibody-induced fetal loss. J. Clin. Invest. 112, 1644–1654. 14660741 10.1172/JCI18817 PMC281643

[R24] Gou SJ, Yuan J, Wang C, Zhao M-H, Chen M, 2013. Alternative complement pathway activation products in urine and kidneys of patients with ANCA-associated GN. Clin. J. Am. Soc. Nephrol. 8, 1884–1891. 10.2215/CJN.02790313 PMC3817906 24115193

[R25] Hakobyan S, Luppe S, Evans DR, , 2017. Plasma complement biomarkers distinguish multiple sclerosis and neuromyelitis optica spectrum disorder. Mult. Scler. 23, 946–955. 27613120 10.1177/1352458516669002

[R26] Hill GS, Delahousse M, Nochy D, , 2001. Predictive power of the second renal biopsy in lupus nephritis: Significance of macrophages. Kidney Int. 59, 304–316. 11135084 10.1046/j.1523-1755.2001.00492.x

[R27] Huang Y-F, Sandholm K, Persson B, Nilsson B, Punga AR, 2024. Visualization and characterization of complement activation in acetylcholine receptor antibody seropositive myasthenia gravis. Muscle Nerve 70, 851–861. 39115039 10.1002/mus.28227

[R28] Humbles AA, Lu B, Nilsson CA, , 2000. A role for the C3a anaphylatoxin receptor in the effector phase of asthma. Nature 406, 998–1001. 10984054 10.1038/35023175

[R29] Iacomino N, Vanoli F, Frangiamore R, , 2022. Complement activation profile in myasthenia gravis patients: Perspectives for tailoring anti-complement therapy. Biomedicines 10. 10.3390/biomedicines10061360 PMC9220000 35740382

[R30] Java A, Kim AHJ, 2023. The role of complement in autoimmune disease-associated thrombotic microangiopathy and the potential for therapeutics. J. Rheumatol. 50, 730–740. 36642429 10.3899/jrheum.220752

[R31] Jayne DRW, Merkel PS, Schall TJ, Bekker P, Group AS, 2021. Avacopan for the treatment of ANCA-Associated Vasculitis. NEJM 384, 599–609. 33596356 10.1056/NEJMoa2023386

[R32] Jia C, Tan Y, Zhao M-H, 2022. The complement system and autoimmune diseases. Chronic Dis. Trans. Med. 8, 184–190. 10.1002/cdt3.24 PMC9481883 36161202

[R33] Johansson L, Berglin E, Eriksson O, Mohammad AJ, Dahlqvist J, Rantapää-Dahlqvist S, 2022. Complement activation prior to symptom onset in myeloperoxidase ANCA-associated vasculitis but not proteinase 3 ANCA associated vasculitis - A Swedish biobank study. Scand. J. Rheumatol. 51, 214–219. 35048784 10.1080/03009742.2021.1989814

[R34] Kallenberg CGM, Heeringa P, 2012. Complement is crucial in the pathogenesis of ANCA-associated vasculitis. Kidney Int. 83, 16–18. 10.1038/ki.2012.371 23271485

[R35] Kamitaki N, Sekar A, Handsaker RE, , 2020. Complement genes contribute sex-biased vulnerability in diverse disorders. Nature 582, 577–581. 32499649 10.1038/s41586-020-2277-x PMC7319891

[R36] Kaya Z, Afanasyeva M, Wang Y, , 2001. Contribution of the innate immune system to autoimmune myocarditis: a role for complement. Nat. Immunol. 2, 739–745. 11477411 10.1038/90686

[R37] Kim AHJ, Strand V, Sen DP, , 2019. Association of blood concentrations of complement split product iC3b and serum C3 with systemic lupus erythematosus disease activity. Arthritis Rheum. 71, 420–430. 10.1002/art.40747 PMC6393208 30294950

[R38] Kitching AR, Anders HJ, Basu N, , 2020. ANCA-associated vasculitis. Nat. Rev. Dis. Primers 6. 10.1038/s41572-020-0204-y 32855422

[R39] Kohl J, 2006. Self, non-self, and danger: a complementary view. Adv. Exp. Med. Biol. 586, 71–94. 16893066 10.1007/0-387-34134-X_6

[R40] Kuhn KA, Cozine CL, Tomooka B, Robinson WH, Holers VM, 2008. Complement receptor CR2/CR1 deficiency protects mice from collagen-induced arthritis and associates with reduced autoantibodies to type II collagen and citrullinated antigens. Mol. Immunol. 45, 2808–2819. 18374982 10.1016/j.molimm.2008.01.036

[R41] Kulik L, Laskowski J, Renner B, , 2019. Targeting the immune complex-bound complement C3d ligand as a novel therapy for lupus. J. Immunol. 203, 3136–3147. 31732528 10.4049/jimmunol.1900620 PMC6900485

[R42] Le Stang M-B, Gleeson PJ, Daha MR, Monteiro RC, van Kooten C, 2021. Is complement the main accomplice in IgA nephropathy? From initial observations to potential complement-targeted therapies. Mol. Immunol. 140, 1–11. 34601376 10.1016/j.molimm.2021.09.010

[R43] Li NL, Birmingham DJ, Rovin BH, 2021. Expanding the role of complement therapies: the case for lupus nephritis. J. Clin. Med. 10. 10.3390/jcm10040626 PMC7915321 33562189

[R44] Lieberman LA, Mizui M, Nalbandian A, Bossé E, Crispín JC, Tsokos GC, 2015. Complement receptor of the immunoglobulin superfamily reduces murine lupus nephritis and cutaneous disease. Clin. Immunol. 160, 286–291. 25988858 10.1016/j.clim.2015.05.006

[R45] Ling GS, Crawford G, Buang N, , 2018. C1q restrains autoimmunity and viral infection by regulating CD8+ T cell metabolism. Science 360, 558–563. 29724957 10.1126/science.aao4555 PMC6545171

[R46] Macedo ACL, Isaac L, 2016. Systemic Lupus Erythematosus and Deficiencies of early Components of the Complement Classical Pathway. Front. Immunol. 10.3389/fimmu.2016.00055. PMC4764694 26941740

[R47] Manderson AP, Botto M, Walport MJ, 2004. The role of complement in the development of systemic lupus erythematosus. Annu. Rev. Immunol. 22, 431–456. 15032584 10.1146/annurev.immunol.22.012703.104549

[R48] Mastellos DC, Lambris JD, 2025. ‘Complement-ing’ tissue inflammation via granzyme K? Nat. Immunol. 10.1038/s41590-025-02120-y. 40186070

[R49] Mastellos DC, Blom AM, Connolly ES, , 2019. ‘Stealth’ corporate innovation: an emerging threat for therapeutic drug development. Nat. Immunol. 20, 1409–1413. 31562490 10.1038/s41590-019-0503-1 PMC7368001

[R50] Matsushita M, Endo Y, Fujita T, 2000. Cutting edge: complement-activating complex of ficolin and mannose-binding lectin-associated serine protease. J. Immunol. 164 (5), 2281–2284. 10679061 10.4049/jimmunol.164.5.2281

[R51] McGaha TL, Madaio MP, 2014. Lupus nephritis: animal modeling of a complex disease syndrome pathology. Drug Disc. Today Dis. Models 11, 13–18. 10.1016/j.ddmod.2014.08.002 PMC4337231 25722732

[R52] Miwa TLZ, Maldonado MA, Madaio MP, Eisenberg RA, Song W-C, 2012. Absence of CD59 exacerbates systemic autoimmunity in MRL/lpr mice. J. Immunol. 189, 5434–5441. 23109726 10.4049/jimmunol.1201621 PMC3507618

[R53] Miyakawa Y, Yamada H, Kosaka K, 1981. Defective immune-adherence (C3b) receptor on erythrocytes from patients with systemic lupus erythematosus. Lancet 2, 493–497. 6115248 10.1016/s0140-6736(81)90882-5

[R54] Morgan BP, Harris CL, 2015. Complement, a target for therapy in inflammatory and degenerative diseases. Nat. Rev. Drug Discov. 14, 857–877. 26493766 10.1038/nrd4657 PMC7098197

[R55] Morgan BP, Chamberlain-Banoub J, Neal JW, Song W, Mizuno M, Harris CL, 2006. The membrane attack pathway of complement drives pathology in passively induced experimental autoimmune myasthenia gravis in mice. Clin. Exp. Immunol. 146, 294–302. 17034582 10.1111/j.1365-2249.2006.03205.x PMC1942050

[R56] Morgan BP, Boyd C, Bubeck D, 2017. Molecular cell biology of complement membrane attack. Semin. Cell Dev. Biol. 72, 124–132. 28647534 10.1016/j.semcdb.2017.06.009

[R57] Morgan BP, Gommerman JL, Ramaglia V, 2021. An “outside-in” and “inside-out” consideration of complement in the multiple sclerosis brain: Lessons from development and neurodegenerative diseases. Front. Cell. Neurosci. 10.3389/fncel.2020.600656. PMC7817777 33488361

[R58] Muller-Eberhard HJ, 1988. Molecular organization and function of the complement system. Annu. Rev. Biochem. 57, 321–347. 3052276 10.1146/annurev.bi.57.070188.001541

[R59] Nakano S, Engel AG, 1993. Quantitative immunocytochemical analysis of inflammatory cells and detection of complement membrane attack complex at the end-plate in 30 patients. Neurology 43. 10.1212/wnl.43.6.1167 8170563

[R60] Nelson RA, 1953. The immune-adherence phenomenon. Science 118, 733–737. 13122009 10.1126/science.118.3077.733

[R61] Nilsson B, Ekdahl KN, 2012. Complement diagnostics: concepts, indications, and practical guidelines. Clin. Dev. Immunol. 2012, 962702. 10.1155/2012/962702 (Epub 2012 Nov 14). 23227092 PMC3511841

[R62] Noris M, Caprioli J, Bresin E, , 2010. Relative role of genetic complement abnormalities in sporadic and familial aHUS and their impact on clinical phenotype. Clin. J. Am. Soc. Nephrol. 5, 1844–1859. 20595690 10.2215/CJN.02210310 PMC2974386

[R63] Oba R, Kanzaki G, Sasaki T, , 2021. Long-term renal survival in antineutrophil cytoplasmic antibody–associated glomerulonephritis with complement C3 deposition. Kidney Intl. Rep. 6, 2661–2670. 10.1016/j.ekir.2021.08.005 PMC8484117 34622105

[R64] Pandey S, Maharana J, Li XX, Woodruff TM, Shukla AK, 2020. Emerging insights into the structure and function of complement C5a receptors. Trends Biochem. Sci. 45, 693–705. 32402749 10.1016/j.tibs.2020.04.004

[R65] Parikh SV, Malvar A, Song H, , 2017. Molecular imaging of the kidney in lupus nephritis to characterize response to treatment. Transl. Res. 182, 1–13. 27842222 10.1016/j.trsl.2016.10.010 PMC5362303

[R66] Paronetto F, Koffler D, 1965. Immunofluorescent localization of immunoglobulins, complement, and fibrinogen in human diseases. I. Systemic lupus erythematosus. J. Clin. Invest. 44, 1657–1664. 4158434 10.1172/JCI105272 PMC292650

[R67] Peffault de Latour R, Röth A, Kulasekararaj AG, , 2024. Oral iptacopan monotherapy in paroxysmal nocturnal hemoglobinuria. NEJM 390, 994–1008. 38477987 10.1056/NEJMoa2308695

[R68] Penfold RS, Prendecki M, McAdoo S, Tam FWK, 2018. Primary IgA nephropathy: current challenges and future prospects. Int. J. Neph. Renovasc. Dis. 11, 137–148. 10.2147/IJNRD.S129227 PMC5905843 29695925

[R69] Perkovic V, Barratt J, Rovin B, , 2025. Alternative complement pathway inhibition with iptacopan in IgA nephropathy. NEJM 392, 531–543. 39453772 10.1056/NEJMoa2410316

[R70] Pittock SJ, Lennon VA, McKeon A, , 2013. Eculizumab in AQP4-IgG-positive relapsing neuromyelitis optica spectrum disorders: an open-label pilot study. Lancet Neurol. 12, 554–562. 23623397 10.1016/S1474-4422(13)70076-0

[R71] Poppelaars F, Goicoechea de Jorge E, Jongerius I, , 2021a. A family affair: addressing the challenges of factor H and the related proteins. Front. Immunol. 10.3389/fimmu.2021.660194 eCollection 2021. PMC8044877 33868311

[R72] Poppelaars F, Faria B, Schwaeble W, Daha MR, 2021b. The contribution of complement to the pathogenesis of IgA Nephropathy: are complement-targeted therapies moving from rare disorders to more common diseases? J. Clin. Med. 10. 10.3390/jcm10204715 PMC8539100 34682837

[R73] R. A, Banda N, Szakonyi G, Chen XS, Holers VM., 2013. Human complement receptor 2 (CR2/CD21) as a receptor for DNA: implications for its roles in the immune response and the pathogenesis of systemic lupus erythematosus (SLE). Mol. Immunol. 53, 99–110. 22885687 10.1016/j.molimm.2012.07.002 PMC3439536

[R74] Ramsey-Goldman R, Li J, Dervieux T, Alexander RV, 2017. Cell-bound complement activation products in SLE. Lupus Sci. Med. 4 (1), e000236. 29214038 10.1136/lupus-2017-000236 PMC5704741

[R75] Reid KB, Turner MW, 1994. Mammalian lectins in activation and clearance mechanisms involving the complement system. Springer Semin. Immunopathol. 15 (4), 307–326. 8153870 10.1007/BF01837363

[R76] Renner B, Poppelaars F, Laskowski J, , 2023. Noninvasive detection of iC3b/C3d deposits in the kidney using a novel bioluminescent imaging probe. J. Am. Soc. Nephrol. 34, 1151–1154. 36995143 10.1681/ASN.0000000000000129 PMC10356150

[R77] Ricklin D, Mastellos DC, Reis ES, Lambris JD, 2018. The renaissance of complement therapeutics. Nat. Rev. Nephrol. 14, 26–47. 29199277 10.1038/nrneph.2017.156 PMC5805379

[R78] Risitano AM, 2012. Paroxysmal nocturnal hemoglobinuria and other complement-mediated hematological disorders. Immunobiology 217, 1080–1087. 22964233 10.1016/j.imbio.2012.07.014

[R79] Röth A, Barcellini W, D’Sa S, , 2021. Sutimlimab in cold agglutinin disease. NEJM 384, 1323–1334. 33826820 10.1056/NEJMoa2027760

[R80] Sato N, Ohsawa I, Nagamachi S, , 2011. Significance of glomerular activation of the alternative pathway and lectin pathway in lupus nephritis. Lupus 20, 1378–1386. 21893562 10.1177/0961203311415561

[R81] Schifferli JG, Taylor RP, 1989. Physiological and pathological aspects of circulating immune complexes. Kidney Int. 35, 993–1003. 2651776 10.1038/ki.1989.83

[R82] Schifferli J, Ng YC, Paccaud J-P, Walport MJ, 1989. The role of hypocomplementaemia and low erythrocyte complement receptor type 1 numbers in determining abnormal immune complex clearance in humans. Clin. Exp. Immunol. 75, 329–335. 2522842 PMC1541944

[R83] Seiler DL, Kleingarn M, Kähler KH, , 2022. C5aR2 deficiency ameliorates inflammation in murine epidermolysis bullosa acquisita by regulating Fcγ receptor expression on neutrophils. J. Invest. Dermatol. 142, 2715–2723.e2. 35007559 10.1016/j.jid.2021.12.029

[R84] Sekine H, Reilly CM, Molano ID, , 2001. Complement component C3 is not required for full expression of immune complex glomerulonephritis in MRL/lpr mice. J. Immunol. 166, 6444–6451. 11342671 10.4049/jimmunol.166.10.6444

[R85] Shi J, Rose EL, Singh A, , 2014. TNT003, an inhibitor of the serine protease C1s, prevents complement activation induced by cold agglutinins. Blood 123, 4015–4022. 24695853 10.1182/blood-2014-02-556027

[R86] Siegel CH, Sammaritano LR, 2024. Systemic Lupus Erythematosus: A Review. JAMA 331 (17), 1480–1491. 10.1001/jama.2024.2315. 38587826

[R87] Simoni L, Presumey J, van der Poel CE, , 2020. Complement C4A regulates autoreactive B cells in murine lupus. Cell Rep. 33. 10.1016/j.celrep.2020.108330 PMC7927756 33147456

[R88] Suzuki H, Kiryluk K, Novak J, , 2011. The pathophysiology of IgA nephropathy. JASN 22, 1795–1803. 21949093 10.1681/ASN.2011050464 PMC3892742

[R89] Thurman JM, Kulik L, Orth H, , 2013. Detection of complement activation using monoclonal antibodies against C3d. J. Clin. Invest. 123, 2218–2230. 23619360 10.1172/JCI65861 PMC3635726

[R90] Vu T, Meisel A, Mantegazza R, , 2023. Terminal complement inhibitor ravulizumab in generalized myasthenia gravis. NEJM Evidence. 10.1056/EVIDoa2100066. 38319212

[R91] Walport MJ, 2002. Complement and systemic lupus erythematosus. Arth. Res. Ther. 4 (Suppl. 3), S279–S293. 10.1186/ar586 PMC3240161 12110148

[R92] Wang Y, Madri JA, Rollins SA, Chodera A, Matis LA, 1996. Amelioration of lupus-like autoimmune disease in NZB/W F1 mice after treatment with a blocking monoclonal antibody specific for complement component C5. Proc. Natl. Acad. Sci. 93, 8563–8568. 8710910 10.1073/pnas.93.16.8563 PMC38712

[R93] Wang MFM, Yu F, Tan Y, Song D, Zhao M-H, 2012. Serum complement factor H is associated with clinical and pathological activities of patients with lupus nephritis. Rheumatology 51, 2269–2277. 22956549 10.1093/rheumatology/kes218

[R94] Watanabe H, Garnier G, Circolo A, , 2000. Modulation of renal disease in MRL/lpr mice genetically deficient in the alternative complement pathway factor B. J. Immunol. 164 (2), 786–794. 10623824 10.4049/jimmunol.164.2.786

[R95] Wenderfer SE, Ke B, Hollmann TJ, Wetsel RA, Lan HY, Braun MC, 2005. C5a receptor deficiency attenuates T cell function and renal disease in MRLlpr mice. J. Am. Soc. Nephrol. 16, 3572–3582. 16207826 10.1681/ASN.2005040373

[R96] Wenderfer SE, Wang H, Ke B, Wetsel RA, Braun MC, 2009. C3a receptor deficiency accelerates the onset of renal injury in the MRL/lpr mouse. Mol. Immunol. 46, 1397–1404. 19167760 10.1016/j.molimm.2008.12.004 PMC2697606

[R97] West EE, Woodruff T, Fremeaux-Bacchi V, Kemper C, 2024. Complement in human disease: approved and up-and-coming therapeutics. Lancet 403, 392–405. 37979593 10.1016/S0140-6736(23)01524-6 PMC10872502

[R98] Wetsel RA, 1995. Structure, function and cellular expression of complement anaphylatoxin receptors. Curr. Opin. Immunol. 7, 48–53. 7772282 10.1016/0952-7915(95)80028-x

[R99] Wingerchuk DM, Lucchinetti CF, 2022. Neuromyelitis optica spectrum disorder. NEJM 387, 631–639. 36070711 10.1056/NEJMra1904655

[R100] Wu EY, McInnis EA, Boyer-Suavet S, , 2019. Measuring circulating complement activation products in myeloperoxidase- and proteinase 3-antineutrophil cytoplasmic antibody-associated vasculitis. Arthritis Rheum. 71, 1894–1903. 10.1002/art.41011 PMC6817386 31215772

[R101] Xiao H, Schreiber A, Heeringa P, Falk RJ, Jennette JC, 2007. Alternative complement pathway in the pathogenesis of disease mediated by anti-neutrophil cytoplasmic autoantibodies. Am. J. Pathol. 170, 52–64. 17200182 10.2353/ajpath.2007.060573 PMC1762697

[R102] Yao X, Verkman AS, 2017. Marked central nervous system pathology in CD59 knockout rats following passive transfer of Neuromyelitis optica immunoglobulin G. Acta Neuropath Comm. 10.1186/s40478-017-0417-9. PMC5316191 28212662

[R103] Zhao J, Wu H, Khosravi M, , 2011. Association of genetic variants in complement factor H and factor H-related genes with systemic upus erythematosus susceptibility. PLoS Genet. 10.1371/journal.pgen.1002079. PMC3102741 21637784

